# Psychosocial working conditions and the utilization of health care services

**DOI:** 10.1186/1471-2458-11-642

**Published:** 2011-08-11

**Authors:** Sunday Azagba, Mesbah F Sharaf

**Affiliations:** 1 Department of Economics, Concordia University, 1455 de Maisonneuve Blvd. West, Montréal, Quebec, H3G 1M8, Canada

## Abstract

**Background:**

While there is considerable theoretical and empirical evidence on how job stress affects physical and mental health, few studies have examined the association between job related stress and health care utilization. Using data from the Canadian National Population Health Survey from 2000 to 2008, this paper examines the association between stressful working conditions, as measured by the job strain model, and the utilization of health care services.

**Methods:**

A zero inflated negative binomial regression is used to examine the excess health care utilization due to job strain. Separate regressions are estimated for both males and females since studies have shown gender differences in health care utilization.

**Results:**

Estimates for the whole population show that high or medium job strain has a positive and statistically significant association with the number of visits to both a general practitioner (GP) and a specialist (SP). On average, the number of GP visits is up to 26% more (IRR = 1.26, 95% CI = 1.19-1.31) for individuals with high strain jobs compared to those in the low job strain category. Similarly, SP visits are up to 27% more (IRR = 1.27, 95% CI = 1.14-142) for the high strain category. Results are quantitatively similar for males and females, save for medium strain. In general, findings are robust to the inclusion of workplace social support, health status, provincial and occupational-fixed effects.

**Conclusion:**

Job strain may be positively associated with the utilization of health care services. This suggests that improving psychosocial working conditions and educating workers on stress-coping mechanisms could be beneficial for the physical and mental health of workers.

## Background

There is considerable theoretical and empirical evidence on how job stress negatively affects physical and mental health [1]. Surprisingly, the relationship between job stress and health care utilization has received little attention. Stress has been widely cited as "the 20th century epidemic" and a "worldwide epidemic" according to the United Nations and the World Health Organization [2]. In the U.S, 70 percent of employees consider the work place a significant source of stress, and 51 percent report that job stress reduces their productivity [3]. Gibson [4] estimated that the health care utilization induced by stress costs U.S. companies $68 billion annually and reduces their profits by 10 percent. Goetzel et al. [5] find that the health care expenditures of workers who report high levels of stress are 46 percent greater than workers with low levels of stress.

**Figure 1:**
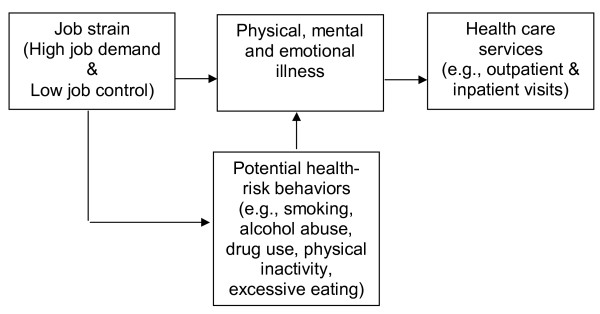
**A simplified model for the relationship between job stress and health care services**. Source: Authors' compilation.

According to Karasek's job strain model, the dominant job stress theory, the combination of on-the-job high psychological demands and low decision latitude lead to physical and mental health problems [6]. Several studies emphasize the importance of including stress as a determinant in models of health service utilization [7-9]. Stress could be linked to increased usage of health care services by a number of routes (see Figure 1). First, individuals may use medical services as a way to cope with stress [10,11]. Second, job stress may cause physical illness, mental and emotional problems all of which increase the demand for health care services. There is medical evidence that stress can adversely affect an individual's immune system, thereby increasing the risk of disease [12,13]. For example, excessive stress has been linked to back pain, colorectal cancer [14], several infectious disease and cardiovascular problems [15,16] and can double the risk of heart attack [17]. Stress may also exacerbate the symptoms of several illnesses including headaches [18-20], diabetes [21], coronary heart disease [22] and upper respiratory infections [13]. Third, job stress may also affect health care usage indirectly by inducing several health risk behaviors such as smoking, drug and alcohol abuse [23,24]. Stress may discourage some healthy behaviors like physical activity, proper diet and it may reduce the consumption of healthy food like fruits and vegetables [25] increase the consumption of fatty and sweet foods [26].

While the association between job stress and health care utilization is theoretically clear, the empirical evidence is very limited. Even the few relevant studies have focused mainly on the effect of major and minor stressful life events and have used small samples that are not necessarily representative of the whole population [8,27]. For example, Brantley et al. [28] examine the ability of minor and major stressful life events to predict medical utilization among 141 low-income, African American family practice patients. They find that minor stressful life events are a significant predictor of outpatient visits but were unrelated to emergency department or inpatient visits. In a related study, Manning et al. [29], using a sample of 260 individuals from two different industries, find that health care claims and costs were positively related to stressful job events and strain. They also find that environmental, stressor and strain variables accounted for up to 16 percent of the variance in health care costs and 21.5 percent of the variance in the number of health care claims.

Health care costs account for a considerable portion of GDP and have shown an upward trend over time in many countries. For example, in Canada expenditure on health care utilization as a percentage of GDP increased from 7% in 1980 to 10.1% in 2007. In the US, total health care expenditure amount to $2.5 trillion, $8,047 per person and this represents 17.3% of the GDP in 2009 [30]. This rose from 9% of GDP in 1980.

Given the recent upward trend of health care costs and the limited empirical research, this study uses nationally representative data from the Canadian National Population Health Survey (NPHS) to examine the association between stressful working conditions and the utilization of health care services. This study is timely and germane to worldwide efforts aimed at curtailing rising health care expenditures.

## Methods

### Data and Variables Description

The data for this study come from the Statistics Canada NPHS household component. NPHS is a nationally representative sample of the Canadian population which collects vital information on health related behavior, as well as corresponding economic and socio-demographic variables. This study uses data from cycle four (2000/01) to cycle eight (2008/09). The sample is restricted to adults aged 18-65 years since a large fraction of those above 65 years are not working. Also, the frailty of health for those over 65 years and unobserved health-related issues may further complicate the results. After excluding missing observations (2,445) and those who are not working (8,237), the final sample includes 29,110 observations. Health services utilization, the dependent variable of interest is measured by the number of visits to (1) a family doctor/general practitioner, and (2) a specialist (excluding eye specialists) during the year preceding the survey interview. Job strain, the main independent variable of interest is an index (score) that is derived by Statistics Canada from job-related questions on decision (control) latitude made up of skill discretion and decision authority, and psychological demands. It is measured as a ratio of psychological demands and decision latitude, where higher values indicate greater job strain. Individuals are stratified based on the distribution of scores into tertiles to represent low (reference category), medium, and high levels of strain.

A number of economic and socio-demographic variables commonly used in the literature are included in the analysis. Age is represented in continuous form. Household income is represented by four dummy variables: low income, middle low income, middle high income (reference category), and high income. Gender is captured by a dummy variable (male = 1, female = 0). Four dummy variables represent individual educational attainment: less than secondary, secondary, some post secondary (reference category), and post secondary. Marital status is represented by three dummy variables: married, separated and single (reference category). Smoking status is classified as: never smoker (reference category), current smoker, and former smoker. Similarly, never drinker (reference category), current drinker, and former drinker represent drinking status. Individual physical activity level is represented by three categories: active, moderate, and inactive (reference category). Ethnicity is captured by a dummy variable (immigrant = 1, Canadian born = 0). A measure of social support in the workplace is included since it has been suggested as an important stress modifier. A higher social support score indicates lower workplace support. Health status is represented by the individual health utility index (HUI) which is a more objective measure than self-rated health. HUI is a comprehensively-scored system for measuring individuals' functional health and a score of 1 indicates perfect health status. It was developed by the Health Utilities Group, McMaster University. The number of chronic diseases for each individual is included and having a regular family doctor is captured by a dummy variable (reg_doc = 1, no reg_doc = 0). Provincial dummy variables are included with British Colombia as the reference category. To control for job-specific effects, seven occupational categories are extracted from the 2007 North American Industry Classification System available in NPHS. An individual's occupation is classified into one of seven groups: mechanical, trade, professional, managerial, health, service, and farm (reference category).

### Statistical Analysis

Multivariate analyses are used to investigate the association between the intensity of health services utilization and job related stress. Given that the outcome measures (GP and SP visits) are positive integer variables (including zeros for non users); count data models are more suitable [31,32]. The benchmark for count data models is a Poisson regression model, which has some restrictive assumptions that are often not satisfied in applied work. For example, a Poisson regression assumes independent count processes, and that the mean and variance are equal (equidispersion). While a negative binomial can correct for overdispersion, unobserved individual heterogeneity due to excess zeros are not well captured. Therefore, a zero-inflated negative binomial regression may be more appropriate. Since negative binomial and the zero-inflated negative binomial are not nested models, the Vuong test is performed to determine the appropriate model. The test results show zero-inflated negative binomial as the preferred model, hence only the zero-inflated negative binomial results are reported.

## Results

**Figure 2:**
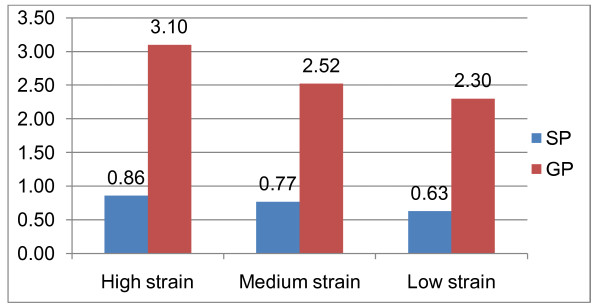
**The unadjusted average health services utilization based on job strain categories**. Source: Authors' calculation based on Canadian National Population Health Survey.

Table 1 reports the summary statistics of the variables included in the analysis. About half (48%) of the sample is female, 63% are married, 47% with post secondary education, 46% are with high income, 85% are non-immigrants, 37% are from Ontario, 48% are physically inactive and 40% are working in mechanical and trade occupations. The average age of individuals in the sample is 40 years. On average, individuals in the sample visit general practitioners and specialists 2.61 and 0.75 times respectively. The average health utility index (0.92) indicates a high health status for the Canadian population. The unconditional analysis of health services utilization according to job strain tertiles are shown in Figure 2. This indicates that individuals in the high and medium strain tertiles use more general practitioner and specialist services than those on the low job strain tertile.

**Table 1:** Summary statistics

Variables	Mean	S.D
**Numerical variables**		
GP	2.610	4.228
SP	0.745	2.558
Age	40.089	12.076
Social support	4.005	1.915
Health utility index(hui)	0.922	0.127
Chronic conditions	1.228	1.346
**Categorical variables**		
High strain	0.328	
Medium strain	0.244	
low strain	0.426	
Male	0.524	
Female	0.476	
Single	0.264	
Married	0.633	
Separated	0.103	
Less than secondary education	0.099	
Secondary education	0.139	
Some post secondary	0.285	
Post secondary	0.475	
Low income	0.034	
Middle low income	0.124	
Middle high income	0.345	
High income	0.497	
Immigrants	0.150	
Non immigrants	0.850	
Never smoker	0.331	
Current smoker	0.261	
Former smoker	0.410	
Never drinker	0.036	
Current drinker	0.884	
Former drinker	0.078	
Regular doctor	0.84	
No doctor	0.151	
Active	0.250	
Moderate	0.273	
Inactive	0.477	
Newfoundland	0.016	
Prince Edwards	0.005	
Nova Scotia	0.030	
New Brunswick	0.024	
Quebec	0.250	
Ontario	0.370	
Manitoba	0.036	
Saskatchewan	0.032	
Alberta	0.113	
British Colombia	0.120	
Mechanical	0.191	
Trade	0.200	
Professional	0.135	
Managerial	0.174	
Health	0.115	
Farm	0.037	
Service	0.144	
*N*	29110	

The statistics are weighted using the NPHS sampling weights. Numbers represent percentage and for some variables they do not add up to 100 per cent because of rounding.

The incidence-rate ratios (IRR) from the multivariate analyses, which are adjusted for potential confounding variables, are presented in Table 2. The first set of analyses (Model 1) is the baseline specification, while the second model includes an additional confounding variable: workplace social support. In model 3, covariates representing: individual's health status, number of chronic conditions, having a family doctor, province and occupational fixed effects are included. In general, the results are qualitatively similar across the different specifications, namely that job strain has a modest and statistically significant association with the utilization of health care services. Using the low job strain as the reference category, the number of GP visits is 26% more (IRR = 1.26, 95% confidence interval [CI] = 1.19-1.31) (model 1) for individuals with high strain jobs. When additional confounding variables are included (see model 3), individuals in jobs with high strain on average have 10% more (IRR = 1.10, 95% CI = 1.05-1.14) GP visits than the low job strain category. Also, being in the medium job strain category increases GP visits by 1.07 (95% CI = 1.02-1.12) and 1.01 (95% CI = 0.97-1.05) in models 1 and 3, respectively. However, model 3 estimate is not statistically significant as 1.0 is included in the CI. Expected SP visits increases by 27% (IRR = 1.27, 95% CI = 1.14-1.42) (model 1) and 14% (IRR = 1.14, 95% CI = 1.04-1.25) (model 3) for the high job strain category compared with the low job strain category. Furthermore, for the medium job strain category, the number of SP visits increases by 15% (IRR = 1.15, 95% CI = 1.04-1.28) in model 1 and 11% (IRR = 1.11, 95% CI = 1.01-1.22) in model 3. These results indicate that high/medium job strain has a statistically significant association with SP visits. In addition to the whole sample estimation, separate analysis is performed for males and females since studies have shown gender differences in health care utilization, and the results are discussed in the next session.

**Table 2:** Zero inflated negative binomial regression: incidence rate ratio (job strain and the use of health services)

	General practitioner visits			Specialist visits		
		
	model(1)	model (2)	model (3)	model(1)	model (2)	model (3)
**Whole population**						
High job strain	1.26*** (1.19-1.31)	1.23*** (1.18-1.29)	1.10*** (1.05-1.14)	1.27*** (1.14-1.42)	1.23*** (1.11-1.37)	1.14*** (1.04-1.25)
Medium job strain	1.07*** (1.02-1.12)	1.06*** (1.02-1.11)	1.01 (0.97-1.05)	1.15*** (1.04-1.28)	1.14*** (1.03-1.26)	1.11** (1.01-1.22)
*N*		29110			29105	
**Males**						
High job strain	1.27*** (1.18-1.38)	1.26*** (1.17-1.36)	1.11*** (1.04-1.19)	1.28*** (1.07-1.54)	1.26*** (1.06-1.50)	1.16* (1.00-1.34)
Medium job strain	1.03 (0.96-1.10)	1.02 (0.95-1.09)	0.96 (0.90-1.02)	1.04 (0.88-1.23)	1.04 (0.88-1.22)	1.00 (0.86-1.17)
*N*		14328			14324	
**Females**						
High job strain	1.24*** (1.17-1.31)	1.23*** (1.16-1.31)	1.10*** (1.05-1.16)	1.27*** (1.13-1.44)	1.23*** (1.08-1.39)	1.13** (1.01-1.26)
Medium job strain	1.11*** (1.04-1.18)	1.10*** (1.04-1.17)	1.06** (1.00-1.12)	1.24*** (1.10-1.39)	1.22*** (1.09-1.37)	1.19*** (1.06-1.33)
*N*		14782			14781	

This table reports estimated coefficients (*β_i_*) of the zero-inflated negative binomial regression transformed to incidence-rate ratios , *** p < 0.01, ** p < 0.05, * p < 0.1, confidence interval at 95% are in parentheses. Model 1 is the baseline specification, while model 2 includes an additional confounding variable, workplace social support. In model 3, covariates representing: individual's health status, number of chronic conditions, having a family doctor, province and occupational fixed effects are included

### Heterogeneous results by gender

Estimates for the association between high job strain and GP and SP services are similar for males and females. For example, for males, GP and SP services utilization increases by 26% (IRR = 1.26) for the high job strain category compared with the low job strain category in model 2. Similarly for females, the excess use of GP and SP services due to high strain is 23% (IRR = 1.23). The association between medium strain and health services utilization is statistically significant only for females. For instance, compared with the low strain category, GP and SP visits increases by 10% (IRR = 1.10, 95% CI = 1.04-1.17) and 22% (IRR = 1.22, 95% CI = 1.09-1.37, respectively.

## Discussion

This study uses a nationally representative data from the Canadian National Population Health Survey to examine the association between job stress and the utilization of health care services. There is substantial evidence that job strain negatively affect physical and mental health whether directly or indirectly. Nonetheless, the literature on the association between job strain and health care utilization is sparse. Even the few relevant studies have focused mainly on the effect of major and minor stressful life events and used small non-generalized samples.

The increasing growth rate of health care spending in many countries is of great importance among academics and policy makers. Countries are experiencing higher spending on health care than the growth rate of their economies. There are concerted efforts aimed at reducing health care cost given the high levels reached in recent times. Stressful working conditions may be associated with higher use of health care services directly by causing physical illness, mental and emotional problems. Medical evidence suggests that stress suppresses the immune system, thereby increasing exposure to several infectious diseases and cardiovascular problems. It may also exacerbate symptoms of several illnesses including headaches [18-20], coronary heart disease [21] and upper respiratory infections [22]. Moreover, stress may increase health care usage indirectly by inducing several health risk behaviors such as smoking [23,24] and discouraging healthy behaviors like physical activity. Individuals may also use medical services as a way to cope with stress [10,11].

Results of both the conditional and unconditional analyses demonstrate that high job strain is associated with higher health care utilization. On average, individuals in jobs with high or medium strain use more health care services than those in jobs with low strain. In particular, the number of GP visits is up to 26% more for individuals with high strain jobs compared to those in the low job strain category. Similarly, SP visits are up to 27% more for the high strain category. In general, the results are robust to the inclusion of individual's health status, number of chronic conditions, having a family doctor, province and occupational fixed effects. Results also show that the medium strain has a statistically significant association with the utilization of health services only for females. This could be due to differences in stress coping abilities between males and females. Research shows that there are gender differences in how males and females perceive and cope with stressful events. It has been argued that males usually tend to use "problem-focused coping" and the "fight-or-flight" response, while females may use "emotion-focused coping" and a "tend-and-befriend" response to stress [33].

It is worth mentioning that the intensity of health care services may be affected by the source of financing for these services. In Canada, the health care system is publicly financed, where citizens and permanent residents are medically covered for inpatient and outpatient visits. Consequently, this may strengthen the association between job strain and the intensity of using outpatient visits.

This study has some limitations. First, though the current study controls for potential confounders that are widely used in the health care utilization literature, there may be other potential confounders for which the study did not control. Second, the outcome variables, SP and GP visits are self reported. However, this is standard in the health care utilization literature. Third, the findings of the current study may not imply causality. Hence, future research using prospective data may be needed to recommend policy changes. Fourth, the current study did not control for stress coping ability, as there is no information available about this in the data set. However, the current study controls for social support, since it has been suggested that stress interacts with other factors in influencing medical utilization. In line with previous studies, the inclusion of the social support index in model 2 reduced the association between job strain and health service usage. Studies have shown that social support reduces strains, mitigates perceived stressors, and moderates the stressor-strain relationship [34]. Pilisuk et al. [35] found that stress increases utilization of outpatient services and that social support helps in reducing this effect. Similar results are found by Counte and Glandon [9].

## Conclusions

We find that high job stress is associated with higher utilization of health care services. The findings of this paper suggest that improving stressful working conditions and educating workers on stress-coping mechanisms may help in reducing health care costs attributable to psychosocial working conditions. The welfare gains from these stress management programs are not limited to reducing health care costs attributable to job stress. Other economic gains, for example, include increased productivity among workers, reduction in absenteeism and employee turnover in addition to other costs borne by employers.

## Competing interests

The authors declare that they have no competing interests.

## Authors' contributions

Both authors contributed equally to this work. Both authors read and approved the final manuscript.

## Pre-publication history

The pre-publication history for this paper can be accessed here:

http://www.biomedcentral.com/1471-2458/11/642/prepub

## References

[B1] Brantley PJ Ames SC Sutker PB, Adams HE Psychobiology of Health and Disease Comprehensive handbook of psychopathology 2001 3 New York, Plenum publishers

[B2] Scott C Optimal stress 2010 John Wiley and Sons Inc. Hoboken New Jersey

[B3] American Psychological Association Stress in America 2009 http://www.apa.org/news/press/releases/stress-exec-summary.pdf

[B4] Gibson V Stress in the workplace: A hidden cost factor HR Focus 1993 70 15

[B5] Goetzel RZ Anderson DR Whitmer RW Ozminkowski RJ Dunn RL Wasserman J The Health Enhancement Research Organization (HERO) Research Committee The relationship between modifiable health risks and health care expenditures: An analysis of the multi-employer HERO health risk and cost database Journal of Occupational and Environmental Medicine 1998 40 10 843 854 10.1097/00043764-199810000-00003 9800168

[B6] Karasek R Theorell T Healthy work: stress, productivity, and the reconstruction of working life 1990 New York: Basic Books

[B7] Mechanic D Correlates of physician utilization: Why do major multivariate studies of physician utilization find trivial psychosocial and organizational effects? Journal of Health and Social Behavior 1979 20 387 396 10.2307/2955413 317291

[B8] Gortmaker SL Eckenrode J Gore S Stress and the utilization of health services:A time series and cross-sectional analysis Journal of Health and Social Behavior 1982 23 24 38 7077069

[B9] Counte MA Glandon GL A panel study of life stress, social support, and the health services utilization of older persons Medical Care 1991 29 348 361 10.1097/00005650-199104000-00004 2020203

[B10] Mechanic D Volkart EH Stress, illness behavior, and the sick role American Sociological Review 1961 26 51 58 10.2307/2090512

[B11] Tessler R Mechanic D Dimond M The effect of psychological distress on physician utilization: A prospective study Journal of Health and Social Behavior 1976 17 353 364 10.2307/2136713 1010916

[B12] Brosschot JF Benschop RJ Godaert G Olff M DeSmet M Heijnen CJ Ballieux RE Influence of life stress on immunological reactivity to mild psychological stress Psychosomatic Medicine 1994 56 216 224 8084967 10.1097/00006842-199405000-00007

[B13] Turner-Cobb JM Steptoe A Psychosocial stress and susceptibility to upper respiratory tract illness in an adult population sample Psychosomatic Medicine 1996 58 404 412 8902892 10.1097/00006842-199609000-00003

[B14] Health Canada Best advice on stress risk management in the workplace Health Canada 2000 http://www.mtpinnacle.com/pdfs/Best-Advise-on-Stress-Management.pdf

[B15] Rosengren A Hawken S Ounpuu S Sliwa K Zubaid M Almahmeed WA Blackett KN Sitthi-amorn C Sato H Yusuf S INTERHEART investigators Association of psychosocial risk factors with risk of acute myocardial infarction in 11119 cases and 13648 controls from 52 countries (the INTERHEART study): case-control study Lancet 2004 364 953 962 10.1016/S0140-6736(04)17019-0 15364186

[B16] Chandola T Britton A Brunner E Hemingway H Malik M Kumari M Badrick E Kivimaki M Marmot M Work stress and coronary heart disease: what are the mechanisms? European Heart Journal 2008 29 640 648 10.1093/eurheartj/ehm584 18216031

[B17] Heart and Stroke Foundation of Canada Report of health Stress threatening Canadians health, Heart and Stroke Foundation warns 2000 http://www.heartandstroke.com/site/apps/nlnet/content2.aspx?c=ikIQLcMWJtE&b=4955951&ct=4512825

[B18] Sorbi MJ Maassen GH Spierings E A time-series analysis of daily hassles and mood changes in the 3 days before the migraine attack Behavioral Medicine 1996 22 102 113 10.1080/08964289.1996.9933771 9116381

[B19] Benedittis GD Lorenzetti A The role of stressful life events in the persistence of primary headache: Major events vs. daily hassles Pain 1992 51 35 42 10.1016/0304-3959(92)90006-W 1454402

[B20] Fernandez E Sheffield J Relative contributions of life events versus daily hassles to the frequency and intensity of headaches Headache 1996 36 595 602 10.1046/j.1526-4610.1996.3610595.x 8990599

[B21] Kramer JR Ledolter J Manos GN Bayless ML Stress and metabolic control in diabetes mellitus: Methodological issues and an illustrative analysis Annals of Behavioral Medicine 2000 22 17 28 10.1007/BF02895164 10892525

[B22] Twisk J Snel J Kemper H van Mechelen W Changes in daily hassles and life events and the relationship with coronary heart disease risk factors: A 2-year longitudinal study in 27-29-year-old males and females Journal of Psychosomatic Research 1999 46 229 240 10.1016/S0022-3999(98)00088-9 10193913

[B23] Cohen S Williamson GM Spacapan S, Oscamp S Perceived stress in a probability sample of the United Statesthe social psychology of health 1988 Newbury Park, CA: Sage 31 67

[B24] American institute of stress http://www.stress.org/topic-effects.htm

[B25] Oliver G Wardle J Perceived effects of stress on food choice Physiology & Behavior 1999 66 3 511 515 10.1016/S0031-9384(98)00322-9 10357442

[B26] Oliver G Wardle J Gibson EL Stress and food choice: a laboratory study Psychosomatic Medicine 2000 62 6 853 865 11139006 10.1097/00006842-200011000-00016

[B27] Williams R Zyzanski SJ Wright AL Life events and daily hassles and uplifts as predictors of hospitalization and outpatient visits Social Science Medicine 1992 34 763 768 10.1016/0277-9536(92)90363-U 1604370

[B28] Brantley PJ Dutton GR Grothe KB Bodenlos JS Howe J Jones GN Minor Life Events as Predictors of Medical Utilization in Low Income African American Family Practice Patients J Behav Med 2005 28 4 395 401 10.1007/s10865-005-9001-z 16049634

[B29] Manning MR Jackson CN Fusilier MR Occupational Stress and Health Care Use Journal of Occupational Health Psychology 1996 1 1 100 109 9547030 10.1037//1076-8998.1.1.100

[B30] Fritze J Medical expenses have very steep rate of growth USA Today 2010 http://www.usatoday.com/news/health/2010-02-04-health-care-costs_N.htm

[B31] Cameron AC Trivedi PK Regression Analysis of Count Data 1998 New York: Cambridge University Press

[B32] Winkelmann R Econometric Analysis of Count Data 2008 5 Springer: Heidelberg, New York

[B33] Taylor SE Klein LC Lewis BP Gruenewald TL Gurung RA Updegraff JA Biobehavioral responses to stress in females: tend-and befriend, not fight-or-flight Psychol Rev 2000 107 411 429 10941275 10.1037/0033-295x.107.3.411

[B34] Viswesvaran C Sanchez JI Fisher J The Role of Social Support in the Process of Job Stress: A Meta-Analysis Journal of Vocational Behavior 1999 54 2 314 334 10.1006/jvbe.1998.1661

[B35] Pilisuk M Boylan R Acredolo C Social support, life stress, and subsequent medical care utilization Health Psychology 1987 6 273 288 3608943 10.1037//0278-6133.6.4.273

